# Characterization of RNA editome in primary and metastatic lung adenocarcinomas

**DOI:** 10.18632/oncotarget.14076

**Published:** 2016-12-21

**Authors:** Lihua Peng, Leo J Lee, Heng Xiong, Hong Su, Junhua Rao, Dakai Xiao, Jianxing He, Kui Wu, Dongbing Liu

**Affiliations:** ^1^ BGI Education Center, University of Chinese Academy of Sciences, Shenzhen 518083, China; ^2^ BGI-Shenzhen, Shenzhen 518083, China; ^3^ Department of Electrical and Computer Engineering, Donnelly Centre for Cellular and Biomolecular Research, University of Toronto, Toronto, Ontario M5S 3G4, Canada; ^4^ Department of Thoracic Surgery, The First Affiliated Hospital of Guangzhou Medical University, Guangzhou 510120, China; ^5^ Guangzhou Institute of Respiratory Disease & State Key Laboratory of Respiratory Disease, Guangzhou 510120, China; ^6^ Research Center for Translational Medicine, The First Affiliated Hospital of Guangzhou Medical University, Guangzhou 510120, China; ^7^ National Clinical Research Center for Respiratory Disease, Guangzhou 510120, China; ^8^ Department of Biology, University of Copenhagen, Copenhagen N DK-2200, Denmark

**Keywords:** lung adenocarcinoma, RNA editing, hyper-editing, primary, metastatic

## Abstract

RNA editing results in post-transcriptional modification and could potentially contribute to carcinogenesis. However, RNA editing in advanced lung adenocarcinomas has not yet been studied. Based on whole genome and transcriptome sequencing data, we identified 1,071,296 RNA editing events from matched normal, primary and metastatic samples contributed by 24 lung adenocarcinoma patients, with 91.3% A-to-G editing on average, and found significantly more RNA editing sites in tumors than in normal samples. To investigate cancer relevant editing events, we detected 67,851 hyper-editing sites in primary and 50,480 hyper-editing sites in metastatic samples. 46 genes with hyper-editing in coding regions were found to result in amino acid alterations, while hundreds of hyper-editing events in non-coding regions could modulate splicing or gene expression, including genes related to tumor stage or clinic prognosis. Comparing RNA editome of primary and metastatic samples, we also discovered hyper-edited genes that may promote metastasis development. These findings showed a landscape of RNA editing in matched normal, primary and metastatic tissues of lung adenocarcinomas for the first time and provided new insights to understand the molecular characterization of this disease.

## INTRODUCTION

Lung adenocarcinoma is a leading cause of cancerous deaths worldwide [1, 2] and great strides have been made to understand its molecular mechanism so that effective cure could be found [3–7]. Traditionally, mutations of the DNA sequence, especially those in cancer driver genes, have been heavily studied to understand the genetic basis of carcinogenesis. However, RNA editing, a post-transcriptional modification event that can flexibly and dynamically change RNA transcripts, could lead to effects similar to genomic mutations and potentially contribute to tumorigenesis as well. In mammals, RNA editing is mainly accomplished by the adenosine deaminase acting on RNA (ADAR) family of enzymes, which modifies adenosine to inosine (A -> I) [8, 9]. RNA editing is enriched in Alu elements [10], which overlaps with many introns and untranslated regions (UTRs) [11], and can modulate RNA structure, RNA splicing and transcript expression [12]. RNA editing has previously been associated with a few cancer types [12–17], such as hepatocellular carcinoma and prostate cancer. Pan-cancer characterization of editing events, including lung adenocarcinoma, has also been carried out in two recent studies [18, 19] which found that RNA editing levels were significantly higher in lung adenocarcinomas than in paired normal tissues. Han et al. also found that *ADAR1* expression levels exhibited significantly higher in lung adenocarcinomas than in normal samples, while *ADAR2* expression levels were in the opposite direction [19]. Moreover, *ADAR1* was reported to be an oncogene undergoing gene amplification-associated activation that affects downstream RNA editing patterns and patient prognosis in non-small-cell lung cancer cells [20]. Although most RNA editing in cancer were likely to be passenger events, a small portion may act as drivers and serve as potential markers for personalized diagnosis and therapy. For example, elevated editing of *AZIN1*^S367G^ in hepatocellular carcinoma has been reported as oncogenic activity thus might be a potential driver in pathogenesis [13].

To our best knowledge, RNA editome of advanced lung adenocarcinomas, especially metastatic lung adenocarcinomas, has not been systematically characterized and the function of RNA editing in lung adenocarcinomas remains largely unknown. Here, we characterized the RNA editing landscape of the matched normal, primary and metastatic tissues from 24 lung adenocarcinoma patients based on whole genome and transcriptome sequencing and detected a large number of putative RNA editing events and hyper-editing sites, i.e., tumor-specific editing sites or editing sites with significantly higher editing levels in tumors than in adjacent normal samples. We also investigated the possible role of these events in primary and metastatic lung adenocarcinomas and provided new insights to understand the molecular characterization of this disease.

## RESULTS

### The landscape of RNA editing in 24 lung adenocarcinoma patients

**Figure 1:**
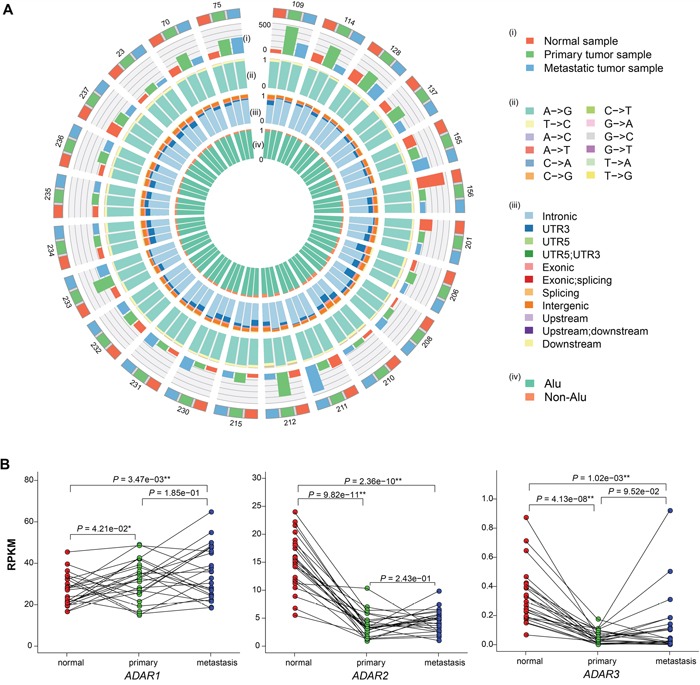
Summary of RNA editing events in adjacent normal, primary and metastatic samples from 24 patients **A**. Circos plot depicting the landscape of RNA editing in lung adenocarcinoma. The outermost circle shows the samples for each patient and the numbers stand for the patient ID. Red, green and blue depict normal, primary and metastatic samples, respectively. The (i) and (ii) circle display the editing rate and the proportion of editing type for each sample, respectively. The (iii) and (iv) circle denote the distribution of editing sites in different genomic regions ((iii): intronic, UTR3, UTR5 etc; innermost: Alu and non-Alu). **B**. Comparison of ADAR family gene expression among adjacent normal, primary and metastatic samples. Red, green and blue points depict normal, primary and metastatic samples, respectively, and lines are drawn to connect samples from the same patient. **, P < 0.01; *, P < 0.05; measured by paired t-test.

The whole genome and transcriptome sequencing data of the adjacent normal, primary and corresponding lymph node metastatic tissues from 24 Chinese lung adenocarcinoma patients were obtained in a previous study [6]. The average depth of whole genome sequencing (WGS) is 31.9×, 49.6× and 51.2× for normal, primary and metastatic tissues, and the transcriptomes were sequenced to ~77M total reads per sample. We used an in-house RNA editing calling pipeline, which is an improved version of a previously published one [21], to identify 1,071,296 RNA editing sites (RES), with an average of 14,879 per sample (range from 800 to 43,708, Supplementary Table 1). As expected, the dominant editing type is A -> G, average 91.3% in each sample (Figure 1A, Supplementary Figure 1A, Supplementary Table 2), and the proportion of this editing type in primary samples were slightly higher than those in normal or metastatic samples (Supplementary Figure 1C). Most of the editing occurred in Alu regions (average 94.5%, range from 88.1% to 98.7%, Figure 1A, Supplementary Figure 1B and Supplementary Figure 1D, Supplementary Table 3). When looking at the distribution of RNA editing across different genomic regions, it most frequently occurred in introns (average 71.5% in each sample, range from 42.9% to 89.5%), followed by 3’ UTR (average 10.5%, range from 2.1% to 33.0%) and intergenic region (average 9.7%, ranger from 4.2% to 15.4%, Figure 1A, Supplementary Figure 1B, Supplementary Table 3).

To account for sequencing and expression differences among samples, we calculated the editing rate (details in Method). The average editing rate is 121.2 (range: 34.1 – 449.5) for normal samples, 190.4 (79.4 – 473.3) for primary samples and 147.2 (62.0 – 448.5) for metastatic samples (Figure 1A, Supplementary Table 1). Comparing the editing rates among the three groups, the editing rates in primary and metastatic samples are significantly higher than those in normal samples (*P* = 3.26e-03 and *P* = 3.46e-02 respectively, Wilcoxon signed rank test). Moreover, the editing rates in primary samples are significantly higher than those in metastatic samples (*P* = 2.45e-02, Wilcoxon signed rank test). However, no correlation was found between the editing rate and clinical information of patients or the number of SNVs (as analyzed in the previous study, Supplementary Figure 2) or tumor purity (average 0.54 in primary and 0.43 in metastatic samples, Supplementary Figure 3).

ADAR family of enzymes was found to be the primary modulator of the frequency and number of sites edited in some cancers [18, 19] and *ADAR1/2* dysregulation demonstrated extremely poor prognoses in non-small-cell lung cancer [20]. Therefore, we calculated the expression of ADAR family of enzymes to investigate the correlation with editing rate (Figure 1B). Compared to normal samples, *ADAR1* was expressed significantly higher in primary and metastatic samples (*P* = 4.21e−02 and *P* = 3.47e−03, paired t-test), while the opposite was found for *ADAR2* and *ADAR3* (*P* = 9.82e−11 and *P* = 4.13e−08 in primary, *P* = 2.36e−10 and *P* = 1.02e−03 in metastatic, paired t-test). The higher expression levels of *ADAR1*, which was also reported in other solid tumors [19, 22–24], may explain why more RNA editing events occurred in tumor samples than in normal samples. However, there is no significant difference of *ADAR1* expression levels between primary and metastatic tumor samples, and the higher RNA editing rate in primary tumor samples could be partly due to the different tissue composition of primary and metastatic samples.

### Detection of hyper-editing events

To further investigate cancer relevant editing events, we detected hyper-editing sites (tumor-specific editing sites, which only edited in tumor but not in adjacent normal, or sites with significantly higher editing levels in tumor than adjacent normal from a patient). In total, we identified 67,851 hyper-editing sites in primary tumors (hyper-PT, Supplementary Table 4) and 50,480 hyper-editing sites in metastatic tumors (hyper-MT, Supplementary Table S). These hyper-editing sites exhibited similar distribution to overall RES in terms of editing type and genomic regions (Supplementary Figure 4 versus Figure 1A, Supplementary Figure 5 and Supplementary Figure 6 versus Supplementary Figure 1). To account for possible sequencing and expression differences among samples, we also calculated a hyper-editing rate and found that hyper-editing rates of hyper-PT (average 31.3) were significantly higher than those of hyper-MT (average 22.1, *P* = 2.29e-02, Wilcoxon signed rank test, Supplementary Figure 4) as well.

### Hyper-editing in CDS regions

**Figure 2:**
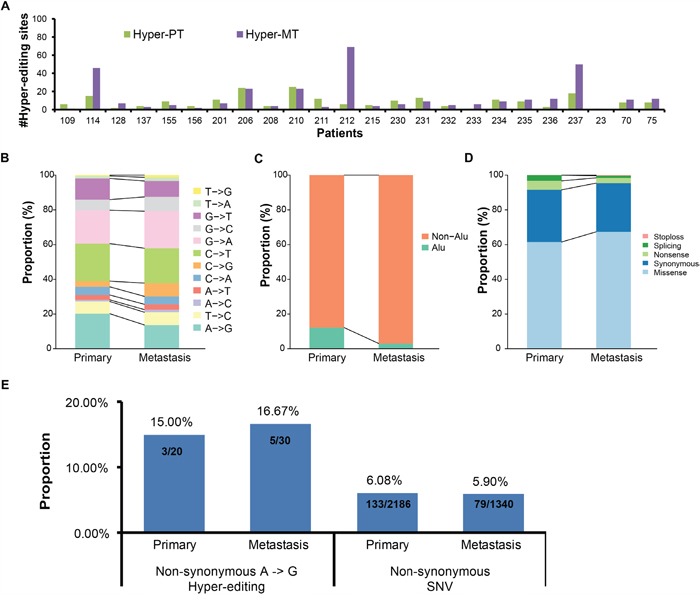
Comparison of hyper-editing sites in CDS regions between primary and metastatic samples **A**. The distribution of hyper-editing sites in CDS regions in each sample. **B-D**. Histograms showing the proportion of editing types (B), the proportion of Alu region (C) and the proportion of mutation types (D) of hyper-editing sites in CDS regions between primary and metastatic samples. **E**. The proportion of the cancer-related genes with non-synonymous A -> G hyper-editing sites or with non-synonymous SNV.

We first analyzed editing in CDS regions (including core splice sites), which may change the translated amino acid sequences. Altogether, we found 213 hyper-PT and 328 hyper-MT in CDS regions. Comparing the number of hyper-PT and hyper-MT in CDS for the same patient, we found them to be similar for most patients, except three (patient ID 114, 212, 237, Figure 2A). Moreover, there was no obvious difference in the proportion of editing type and genomic distribution between hyper-PT and hyper-MT in CDS (Figure 2B, 2C and 2D). Owing to the lack of sufficient validation on non-A->G editing sites reported in previous studies [21, 22], we only included A -> G hyper-PT and hyper-MT in subsequent analyses.

In total, we found 46 genes with hyper-editing had non-synonymous changes, including 20 genes with A -> G hyper-PT and 30 genes with A -> G hyper-MT (only 4 genes were shared, Supplementary Table 4, 6 and 8, Supplementary Figure 7). Among them, the editing sites chr8:103841636 in *AZIN1* (S367G) and chr4:2940026 in *NOP14* (I779V), which were previously reported to have significantly elevated editing levels in lung adenocarcinoma from a pan-cancer analysis [19], were detected in two tumors and one tumor, respectively (Table 1). To investigate the role of non-synonymous A -> G hyper-editing, we further compared the proportion of cancer-related genes with non-synonymous A -> G hyper-editing to those with non-synonymous SNVs. We found 15.0% and 16.7% cancer-related genes with non-synonymous hyper-editing in primary and metastatic samples (Table 1), while the proportion of cancer-related genes with non-synonymous SNVs is 6.1% and 5.9% (Figure 2E). This indicates that non-synonymous hyper-editing may play as important a role as non-synonymous SNVs in lung adenocarcinoma, if not more.

**Table 1:** The selected genes with non-synonymous A -> G hyper-editing

Gene Name	Chr:Pos	Editing type	Allele change	Amino acid change	Sample ID	Class	Description
*AZIN1*	chr8:103841636	A->G	c.A1099G	p.S367G	137F, 212G	Reported	Antizyme Inhibitor 1
*NOP14*	chr4:2940026	A->G	c.A2335G	p.I779V	212F	Reported	NOP14 nucleolar protein
*CHD6*	chr20:40127981	A->G	c.A869G	p.D290G	206F	CRRG	Chromodomain helicase DNA binding protein 6
*TCF3*	chr19:1615370	A->G	c.A1736G	p.H579R	211F	CGC	Transcription factor 3
*ATP1A1*	chr1:116931630	A->G	c.A743G	p.N248S	114F, 114G	CGC	ATPase, Na+/K+ transporting, alpha 1 polypeptide
*KDM2A*	chr11:67017933	A->G	c.A1115G	p.H372R	235G	CRRG	Lysine (K)-specific demethylase 2A
*CSF3R*	chr1:36939477	A->G	c.A373G	p.I125V	128G	CGC	Colony stimulating factor 3 receptor (granulocyte)
*MSH2*	chr2:47656925	A->G	c.A1121G	p.Q374R	212G	CGC	MutS Homolog 2
*NUMA1*	chr11:71725974	A->G	c.A2575G	p.I859V	210G	CGC	Nuclear mitotic apparatus protein 1

Note: Cancer Gene Census (CGC); Chromatin remodeling related genes (CRRG). “Reported” stands for gene has been previously reported. Cancer-related genes were found from GCG or TCGA driver genes list or CRRG list.

Interestingly, we found that four cancer-related genes (*CSF3R*, *MSH2*, *NUMA1* and *KDM2A*) only hyper-edited in metastatic but not in primary samples (Table 1), suggesting that these alterations may promote metastasis development. Of which, *CSF3R*, which is a member of the family of cytokine receptors and may also function in some cell surface adhesion or recognition processes, was reported to be related to tumor growth and metastasis [25]. *MSH2*, a component of the post-replicative DNA mismatch repair system, frequently mutated in melanoma brain metastases but not in primary melanomas, indicating the high genomic instability of metastatic samples [26]. Moreover, *MLH1*/*MSH2* defects were strongly associated with a decreased likelihood of lymph node and distant organ metastases in sporadic colorectal cancer [27]. To further investigate the role of non-synonymous hyper-editing genes, we performed pathway enrichment analysis of these genes and found 3 genes (*CSF3R*, *IL12RB1* and *STAT2*) in Jak-STAT signaling pathway only hyper-edited in metastatic but not in primary samples (Supplementary Figure 8, Supplementary Table 9). Activated Jak-STAT pathway could promote cellular invasion and migration in cancer, such as in hepatocellular carcinoma [28] and head and neck squamous cell carcinoma [29]. Some drugs have been reported to decrease proliferation and metastatic behavior of tumor cells by modulating Jak-STAT signaling pathway, such as guggulsterone for pancreatic cancer [30].

### Hyper-editing in non-coding regions

**Figure 3:**
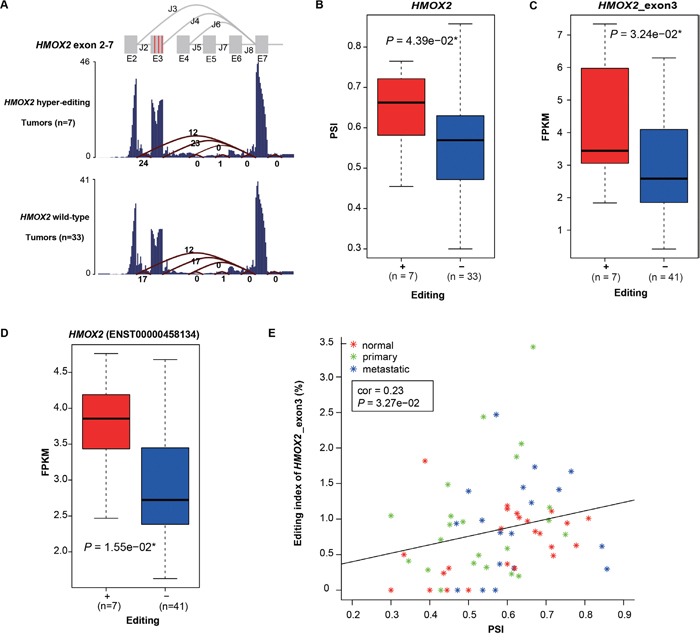
Hyper-editing induced splicing aberration of HMOX2 **A**. The average RNA-seq read coverage and junction counts are shown. Three red vertical lines in exon3 represent three A -> G hyper-editing sites (from left to right, 4533677, 4533713 and 4533730 in chr16) predicted to affect splicing. Samples with the total number of the splice junction reads supporting J2, J3 and J4 less than 10 are excluded. **B** and **C**. Comparison of PSI (B) and expression of exon3 (C) in *HMOX2* between two subgroups: tumor samples with at least one of the three hyper-editing sites in *HMOX2* (“+”) versus those with none (“-”). Wilcoxon rank sum test was used. **D**. Box plot showing the expression levels of the corresponding transcript between “+” and “-” groups. P value was calculated by t-test. **E**. The correlation between the editing index of exon 3 and PSI. Pearson's product-moment correlation was used.

In our samples, 95.7% of RNA editing took place in non-coding regions, especially introns and UTRs, where they could modulate splicing or RNA structure or gene expression. To investigate the potential correlation between editing and RNA splicing, we first used SPIDEX [31] to predict A -> G hyper-editing sites that may influence splicing. 20 A -> G hyper-PT and 16 A -> G hyper-MT were found, with 77.8% occurring in non-coding regions (Supplementary Table 10, Supplementary Figure 7). Of which, *HMOX2* (Heme Oxygenase 2), which belongs to the heme oxygenase family and is related to heme catabolism, was most frequently edited (detected in four primary and three metastatic samples) and harbored three hyper-editing sites (chr16: 4533677, 4533713, 4533730, all located in 5’ UTR of exon3). To investigate the relationship between these three hyper-editing sites and the splicing patterns of *HMOX2* (Figure 3A), we calculated the percent spliced in (PSI, detailed in Method) of exon 3 in *HMOX2*. We found that the PSIs of tumor samples with at least one of three hyper-editing sites were significantly higher than those without them (*P* = 4.39e−02, Wilcoxon signed rank test, Figure 3B). Moreover, both the expression of exon3 and the transcript that contains exon3 (ENST00000458134) exhibited the same pattern (*P* = 3.24e−02 and *P* = 1.55e−02, Wilcoxon signed rank test, Figure 3C and 3D). In addition, we found a weak positive correlation between PSI and the editing index (see Method) of exon3 (Pearson correlation = 0.23, Figure 3E). These results suggested that hyper-editing in 5’ UTR of exon3 could increase the relative usage of exon3 and the expression of the corresponding transcript, and the higher editing index of exon3, the greater the effect.

**Figure 4:**
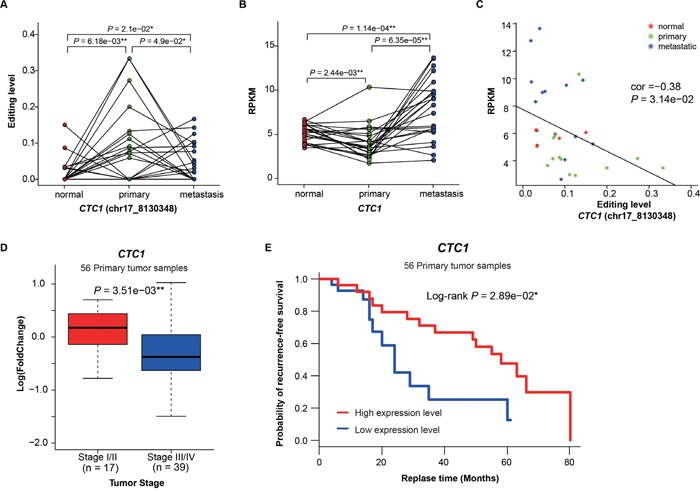
Functional analysis of CTC1 with hyper-editing **A**. The editing levels of the deleterious site (chr17:8130348) in *CTC1* among normal, primary and metastatic samples. **B**. The expression of *CTC1* among three sample groups. **C**. The correlation between the editing level of chr17:8130348 and the expression of *CTC1*. **D**. *CTC1* expression versus tumor stages (*P* value was calculated by the t-test). **E**. Kaplan-Meier survival curves showing the relationship between recurrence-free survival probability and *CTC1* expression (low: less than median; high: greater than median).

To further discover functional editing sites in non-coding regions, we applied CADD [32] to predicted deleterious editing events. 32 A -> G hyper-editing events were predicted to be deleterious, including 14 hyper-PT and 18 hyper-MT, with 59.4% in non-coding regions (Supplementary Table 11, Supplementary Figure 7). Of which, chr17:8130348, located in the 3’ UTR of *CTC1* (CTS Telomere Maintenance Complex Component 1), is the most frequent deleterious hyper-editing site (detected in four primary and one metastatic samples). *CTC1* is a component of the CST complex and this complex plays an essential role in protecting telomeres from degradation. To investigate the role of this event, we first compared the editing level of chr17:8130348 among three sample groups, and found that the editing levels in tumor samples were significantly higher than those in normal samples (*P* = 6.18e−03 and *P* = 2.10e−02), while the editing levels in primary samples were also higher than metastatic samples (*P* = 4.90e−02, paired t-test, Figure 4A). Interestingly, the expression of *CTC1* in primary samples were significantly lower than normal and metastatic samples (*P* = 2.44e−03 and *P* = 6.35e−05, paired t-test, Figure 4B). Moreover, a negative correlation between the editing level of chr17:8130348 and the expression of *CTC1* were found (Pearson correlation = -0.38, Figure 4C), which suggests possible regulation of decreasing *CTC1* expression through RNA editing. Among cancer patients, those of early stage showed higher *CTC1* expression than those of late stage (*P* = 3.51e−03, t-test, Figure 4D). Clinically, *CTC1* down-regulation demonstrated poor recurrence-free survival probability (Log-rank P = 2.89e−02, Figure 4E). These results suggest that when deleterious hyper-editing do not alter amino acid sequences, they may affect gene expression and consequently promote tumor progression or influence clinical outcomes in lung adenocarcinoma.

**Figure 5:**
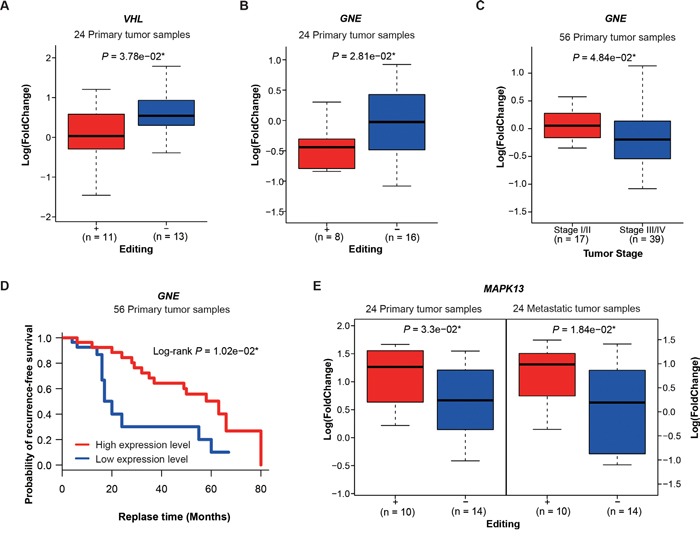
Hyper-editing and differential gene expression **A**. Differential expression of *VHL* between edit+ and edit- primary samples. **B**. Differential expression of *GNE* between edit+ and edit- primary samples. **C**. *GNE* expression versus tumor stages **D**. Kaplan-Meier survival curves showing the relationship between recurrence-free survival probability and *GNE* expression. **E**. Differential expression of *MAPK13* in edit+ and edit- groups for primary and metastatic samples. P values are calculated by t-test.

As the above results showed, editing in UTRs, which contain binding sites for regulatory proteins and microRNAs, may induce differential gene expression in lung adenocarcinoma. To further investigate the association between hyper-editing events in UTRs and gene expression, we classified samples into two subgroups: edit+, which contains any A -> G hyper-editing sites in UTRs of a given gene; edit-, contrasting to edit+. Then we calculated the significance of differential expression between the samples of edit+ and edit- for each gene, requiring each group to have at least four samples. In total, we identified 39 and 30 genes having significant expression differences in primary and metastatic samples, respectively (using a threshold of *P* <= 0.05, t-test, Supplementary Table 12). Among the differentially expressed genes in primary samples, 7 of 39 genes (*VHL*, *GNE*, *POLH*, *MAPK13*, *NOP14*, *INADL*, *CDC5L*) were reported to be cancer-related in the literature (Supplementary Figure 9, Supplementary Table 13). Of which, *VHL* (Von Hippel-Lindau syndrome) is a tumor suppressor and expressed significantly lower in edit+ primary samples than in edit- samples (*P* = 3.78e−02, t-test, Figure 5A). Abnormality of the *VHL* has been reported to be linked to the development of renal carcinomas [33, 34] and hemangioblastomas [35]. In addition, *GNE* (Glucosamine (UDP-N-Acetyl)-2-Epimerase/N-Acetylmannosamine Kinase) expressed significantly lower in edit+ primary samples than edit- samples (*P* = 2.81e−02, t test, Figure 5B). Clinically, we found patients with early stage showed higher *GNE* expression than those with late stage (*P* = 4.84e−02, t-test, Figure 5C). We also found patients with low *GNE* expression levels had poor recurrence-free survival probability (Log-rank *P* = 1.02e−02, Figure 5D). *GNE* dysregulation occurred predominantly in pancreatic cancer but also in other malignancies [36]. Among the differentially expressed genes in metastatic samples, we found 7 of 30 genes (*MAPK13*, *XIAP*, *AHR*, *SPN*, *TER1*, *EMP2*, *NDUFC2*) were cancer-related (Supplementary Figure 10, Supplementary Table 13). Of which, only one, *MAPK13* (Mitogen-Activated Protein Kinase 13), was expressed significantly higher in both edit+ primary and metastatic samples than in corresponding edit- samples (Figure 5E, *P* = 3.3e−02 and *P* = 1.84e−02, t-test). *MAPK13* is a component of the mitogen-activated protein (MAP) kinase family and plays an important role in the development of cancer, such as cholangiocarcinoma [37]. The remaining metastasis-specific genes could be related to metastasis development. Indeed, we found that *XIAP* (X-linked inhibitor of apoptosis), expressed significantly lower in edit+ than in edit- metastatic samples (*P* = 3.27e−02, t-test, Supplementary Figure 10), were reported to promote metastasis or tumor recurrence in hepatocellular carcinoma [38] and in papillary thyroid carcinoma [39], and *AHR*, expressed significantly higher in edit+ than in edit- metastatic samples (*P* = 5.7e−03, t-test, Supplementary Figure 10), were also reported to be associated with metastasis and may be a potential therapeutic target in the treatment of metastatic breast cancer [40].

## DISCUSSION

The pathogenicity of advanced lung adenocarcinomas, especially metastatic lung adenocarcinomas, remains largely unknown and RNA editing is an important source of transcriptome diversification in cancer. This study represents a first systematic investigation and characterization of RNA editome in primary and metastatic lung adenocarcinomas based on matched whole genome and transcriptome sequencing data, where we identified 1,071,296 RES, 67,851 hyper-PT and 50,480 hyper-MT sites. We found that editing events played a role in lung adenocarcinomas via changing amino acids, modulating alternative splicing patterns, such as in 5’ UTR of *HMOX2*, or affecting the expression of genes related to tumor stage and recurrence, such as in 3’ UTRs of *CTC1* and *GNE*. Furthermore, by investigating hyper-edited genes only appeared in metastatic but not primary samples, we discovered genes and pathways that contribute to metastasis development, such as *MSH2* and *XIAP*.

The role of editing events in cancer is still somewhat controversial and further investigations are warranted. One possible role of editing is to regulate alternative splicing. Alternative splicing and RNA editing, which are different post-transcriptional events, have the potential to generate many different mRNAs from the same gene, thus increasing the transcriptome and proteome diversity [41]. Editing could modulate alternative splicing by multiple means: creating the canonical 5’ and 3’ dinucleotide recognition sequences, destroying the 3’ recognition sequence or the internal branch point adenosine, targeting splicing enhancer or suppressor sequences [12]. Currently, little is known about this regulatory mechanism in cancer. A study identified that RNA editing can serve as a mechanism for regulating alternative splicing of *ADAR2* in rat [42]. Evidence also exists to indicate a relationship between *TPH2* (Tryptophan hydroxylase 2) alternative splicing and RNA editing in psychiatric diseases [43]. In acute myeloid leukemia, editing in the putative branch site of tumor suppressor *PTPN6* mRNA, which leads to aberrant intron retention, was suggested to account for *PTPN6* functional insufficiency [44]. In this study, we also found that tumors with hyper-editing events in *HMOX2* were related to the relative usage of exon and the expression of the corresponding transcript. Although there are no studies showing the relationship between *HMOX2* and cancer yet, *HMOX1*, a paralog of *HMOX2*, was known as a poor prognostic predictor and its over-expression may increase the metastatic potential of non-small cell lung cancer [45]. Interaction between RNA editing and alternative splicing, which can create a multitude of functionally distinct protein isoforms, might play a crucial role in tumorigenesis and joint analysis of both processes could be a new trend of cancer research.

RNA editing is dynamic and flexible and the editing levels may vary over time and tumor stages, which could be used to investigate cancer progress [19]. Recently, elevated editing of *AZIN1* in hepatocellular carcinoma was reported to be related to tumor stages [13]. In this study, we compared RNA editome of primary and metastatic samples and found that RNA editing was significantly more frequent in primary than in paired metastatic samples. We also found a few genes affected by hyper-editing could contribute to metastasis development. Future large-scale studies of lung adenocarcinoma samples with more tumor stages and more complete clinical information should help us to better understand the relationship between RNA editing and lung tumor progression. Ultimately, in-depth functional analysis of RNA editing in cancer will further our understanding of the molecular characteristic of this complex disease and may provide new insights for effective personalized therapies.

## MATERIALS AND METHODS

### Sequencing data

We obtained the BWA-aligned DNA sequencing data and RNA sequencing data (fastq files, Illumina sequencing) from the adjacent normal tissue, primary tumor and corresponding lymph node metastases tissue of 24 Chinese lung adenocarcinoma patients in a previous study [6]. The method of sampling and sequencing were described in this previous study.

### Data preprocessing and reads mapping

The raw reads of RNA sequencing data filtering process as follows: (1) Remove reads with sequence adaptors. (2) Remove reads in which unknown bases are more than 10%. (3) Remove low-quality reads, which have >50% in one read. Tophat2 were chose as the mapper for clean RNA-seq reads due to its high accuracy of alignment. After mapping reads to the reference genome (hg19), Picard (v1.84; http://broadinstitute.github.io/picard/) and GATK (v2.8-1) software were used to remove identical reads (PCR duplicates) and recalibrate base quality, respectively.

### In-house RNA editing sites calling pipeline

We used an in-house RNA editing sites calling pipeline, which is an improved version of a previously published one [21], to identify candidate editing sites. The basic principle for identifying an RNA-editing site is that the site must be homozygous for gDNA, while at the same time displaying a mismatch in RNA [46]. Firstly, We summarized the base calls of pre-processed aligned RNA-reads to the human reference in pileup format. Secondly, the initially identified editing sits were then filtered by the following quality-aware steps: (1) The depth, base quality, mapping quality and the frequency of variation were taken into account to do a basic filtering. (2) Statistical test based on binomial distribution B(n, p) were used to distinguish true variants from sequencing errors on every mismatch site [46], where p denotes the background mismatch rate of each transcriptome sequencing, and n denotes sequencing depth on this site. (3) Discard the sites present in dbsnp138. (4) Estimated strand bias and filtered out variants with strand bias based on two-tailed Fisher's exact test. (5) Estimated and filtered out variants with position bias, such as sites at 3’-end or at 5’-end. (6) Discarded the variation site in simple repeat region or homopolymer region or splicing region. Finally, we summarized the candidate editing sites to DNA bam in pileup format, and only the site was homozygous in gDNA will be kept as a true editing site. Ultimately, all true editing sites were met minimal editing level of 10%, minimal edit bases of 3 and minimal depth of 4×.

### Hyper-editing sites and hypo-editing sites calling

For each paired tumor-normal samples, hyper-editing sites were detected in either case as follows. 1) Tumor-specific editing sites, which the sites only edited in tumor but not in adjacent normal from a patient. Namely, a given site only found in tumor samples with a minimal editing level of 10%, minimal edit bases of 3 and minimal depth of 6×, while paired normal samples were covered at least 6× without carrying any edit base. 2) The site showed significantly higher editing level in tumor than adjacent normal from a patient. The difference of editing level must be more than 0.2, and show significantly higher editing level in tumor than in adjacent normal samples (P <= 0.05, fisher test).

Similarly, hypo-editing site was found only in normal samples with a minimal editing level of 10%, minimal edit bases of 3 and minimal depth of 6×, while paired tumor samples were covered at least 6x without carrying any edit base. Or the difference of editing level must be less than -0.2, and show significantly higher editing level in normal than in tumor samples (P <= 0.05, fisher test). The function of hypo-editing sites (Supplementary Table 5 and Supplementary Table 7) were not well known and those sites were ignored in this paper.

### Definition

Editing rate is calculated as the total number of editing sites divided by the total length of transcriptome sequencing (minimal covered depth >=4, in Mega base) for a sample. Hyper-editing rate is defined to be the total number of hyper-editing sites divided by the total length of transcriptome sequencing, while hypo-editing rate was calculated as the total number of hypo-editing sites divided by the total length of transcriptome sequencing.

Editing level of a given editing sites is calculated as the variant-supporting reads divided by the total reads. For each paired tumor-normal samples, the difference in editing level of a given site for a patient is defined to be the editing level of this site in tumor sample minus the editing level in normal sample.

### Gene expression and different expression

RPKM (reads per kilobase per million mapped reads) was used to calculate the gene expression and the formula is shown as follows:

$$\text{RPKM}=\frac{10^{6}C}{NL/10^{3}}$$

In which, *C* is the number of uniquely mapping reads to a given gene, *N* is the total number of reads that are uniquely aligned to all genes and *L* stands for the total length of exons from the given gene.

To measure change in the expression level of a gene, different expression of a given gene was calculated as the log (fold change), and the formula is shown as follows:

$${\text{log }(\text{fold change})=\text{ log}}_{\text{2}}(\frac{RPKM_{tumor}}{RPKM_{normal}})$$

Of which, *RPKM_tumor_* stands for the RPKM of a given gene in tumor sample and *RPKM_normal_* is the RPKM of this gene in normal sample.

### Correlation between editing level and expression of gene

For a given editing site, Pearson's product-moment correlation was used to calculated the correlation between editing level and expression of gene. Samples with covered reads lower than 4 and the editing level equal to zero were excluded from this analysis.

### Editing-induced splicing aberration

We used SPIDEX [31], which a comprehensive set of mutations and their predicted effects on RNA splicing across the entire human genome, to predict the editing sites with effects on splicing. The dataset was downloaded from the website (http://www.deepgenomics.com/spidex-noncommercial-download). We selected the editing sites with the Z-score >= 2 or Z-score <= -2, which were predicted to more higher effect on splicing regulation.

Analysis of splice variants, the splice junction counts of *HMOX2* and the FPKM of exon bins can be performed by SGSeq software package (v1.4.3) available from Bioconductor. We predicted exons and splice junctions from RNA BAM files for given genes using parameters alpha = 1, psi = 0, beta = 0.2, gamma = 0.2.

The percent spliced in (PSI) of *HMOX2* was calculated by the formula as follows:

$$\text{PSI}=\frac{\frac{\left(J2+J4\right)}{2}}{\frac{\left(J2+J4\right)}{2}+J3}$$

In which, *J*2 is the splice junction counts of the junction between exon 2 and exon 3 of *HMOX2*. *J*3 stands for the splice junction counts of the junction between exon 2 and exon 7. *J*4 is the splice junction counts of the junction between exon 3 and exon 7 of this gene.

The editing index of exon 3 in *HMOX2* measures the averaged editing level across exon 3 region, weighted by their expression. It may be quantified by the ratio of the read number of A-to-G/C/T editing sites with effects on splicing to the total number of reads—nucleotides aligned to a genomic adenosine within exon 3 region. Sites with covered reads lower than 4 were excluded from this analysis.

### The deleterious editing sites

Combined Annotation Dependent Depletion (CADD, versions 1.3) is a tool for scoring the deleteriousness of single nucleotide variants in the human genome based on integrating multiple annotations into one metric by contrasting variants that survived natural selection with simulated mutations [32]. The CADD scores data can be downloaded from the website (http://cadd.gs.washington.edu/download). In our work, the editing sites with the PHRED-like scaled C-score >= 20, indicated that these sites are predicted to be the 1% most deleterious substitutions, were detected to be the deleterious editing sites.

### Cancer-related genes list

Cancer-related genes can be found from GCG (Cancer Gene Census) genes list or TCGA driver genes list or chromatin remodeling related genes list. CGC genes [47] can be found in the website (http://cancer.sanger.ac.uk/census) and the TCGA pan-cancer driver genes were found in dataset of a previous paper [48]. All chromatin remodeling related genes were found in three databases: HIstome (The Histone Infobase, http://www.actrec.gov.in/histome/), EpiFactors (http://epifactors.autosome.ru/) and CREMOFAC (Chromatin Remodeling Factors, http://www.jncasr.ac.in/cremofac/menuframe.html) database.

### Pathway enrichment analysis

Editing genes were use to KEGG pathway enrichment analysis by GSEA database (http://www.broadinstitute.org/gsea/index.jsp). Pathways, which the FDR (False discovery rate) q-value was below 0.05, were selected. To further investigate the role of non-synonymous hyper-editing genes, we performed pathway enrichment analysis of these genes. Two pathways were enriched in metastatic samples, while no pathway enriched in primary samples.

### Survival analysis and clinical association analysis

According to the median, we classified 56 primary tumors (including additional 32 primary tumor in Wu et al. paper, the transcriptome data by Ion Proton sequencing) into two groups: high expression level (greater than median) and low expression level (less than median). Kaplan-Meier survival analysis using R package “survival” (http://cran.r-project.org/web/packages/survival/index.html). Log-rank test was used to compare the survival distribution of the above two subgroups.

To analyze the correlation between the expression of a given gene and clinical information, gender, smoking status, age and tumor stages were take into account. T test were used.

## SUPPLEMENTARY MATERIALS FIGURES AND TABLES




