# Supplementary material for: Characterization of RNA editome in primary and metastatic lung adenocarcinomas
Source: Oncotarget. 2016 Dec 21;8(7):11517–29. doi: 10.18632/oncotarget.14076 (PMC5355282; doi:10.18632/oncotarget.14076)
Supplement: Supplementary file 1 [file oncotarget-08-11517-s001.pdf]

Characterization of RNA editome in primary and metastatic lung adenocarcinomas

SUPPLEMENTARY FIGURES AND TABLES

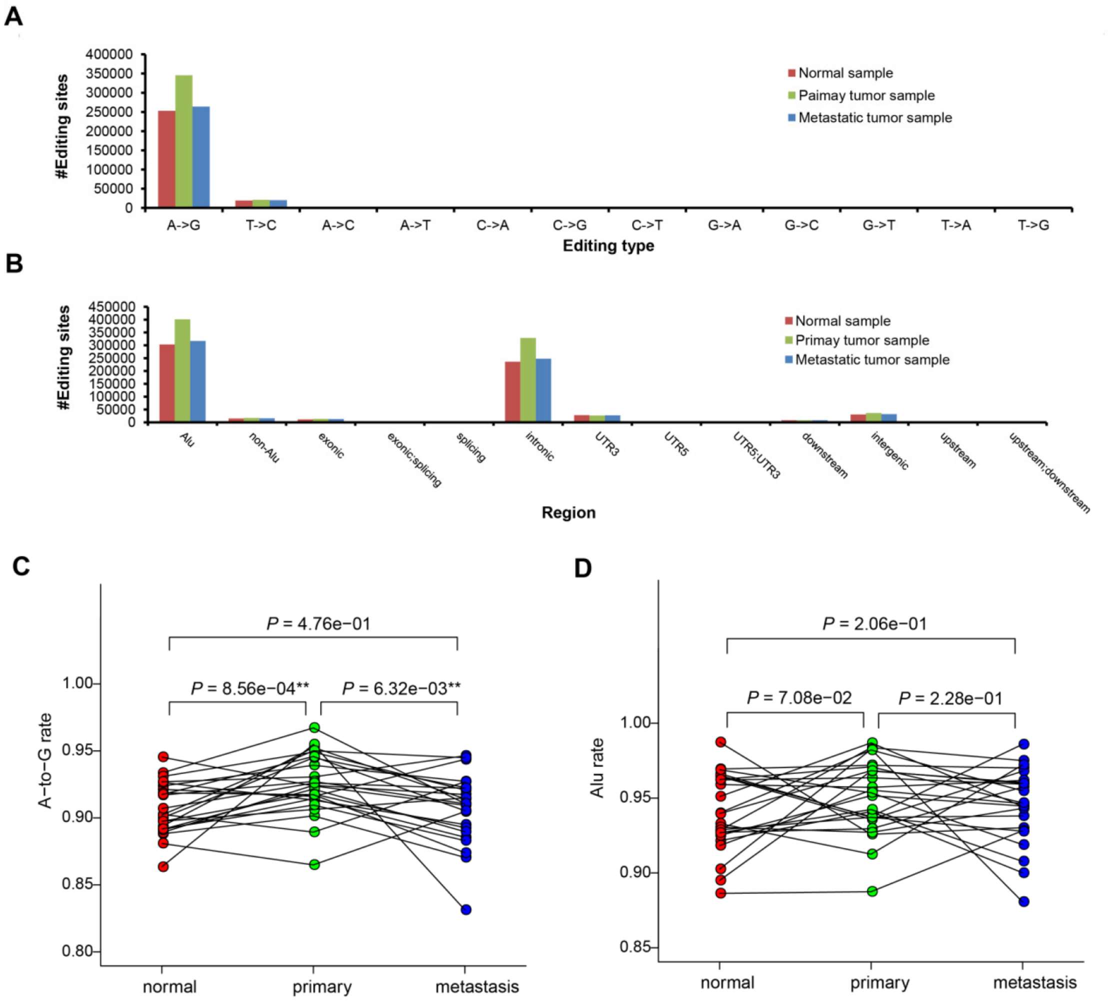

Supplementary Figure 1: Summary of editing type and the distribution of RNA editing sites (RES) in different genomic regions. A. Histogram showing the numbers of editing types of RES in all samples. **B.** Histogram depicting the numbers of RES occur in different regions across genome. **C** and **D.** Comparison of A -> G editing sites rates (C) or the proportion of RES in Alu region (D) among adjacent normal, primary, metastatic samples. **, $P < 0.01$; *, $P < 0.05$; measured by paired t-test.

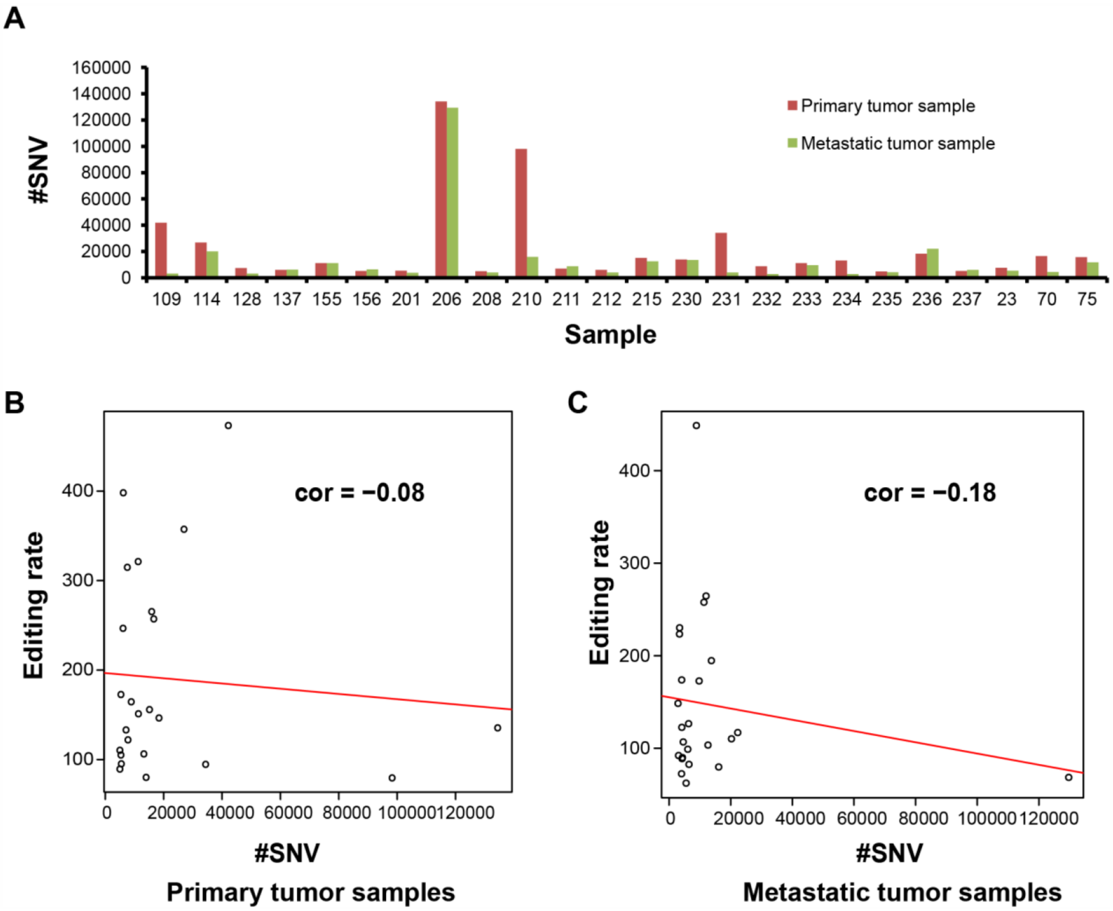

Supplementary Figure 2: Comparison of the number of SNVs and editing rate. A. The distribution of SNV for each samples from 24 patients. **B** and **C.** The relationship between the editing rate and SNV in primary samples (B) and in metastatic samples (C).

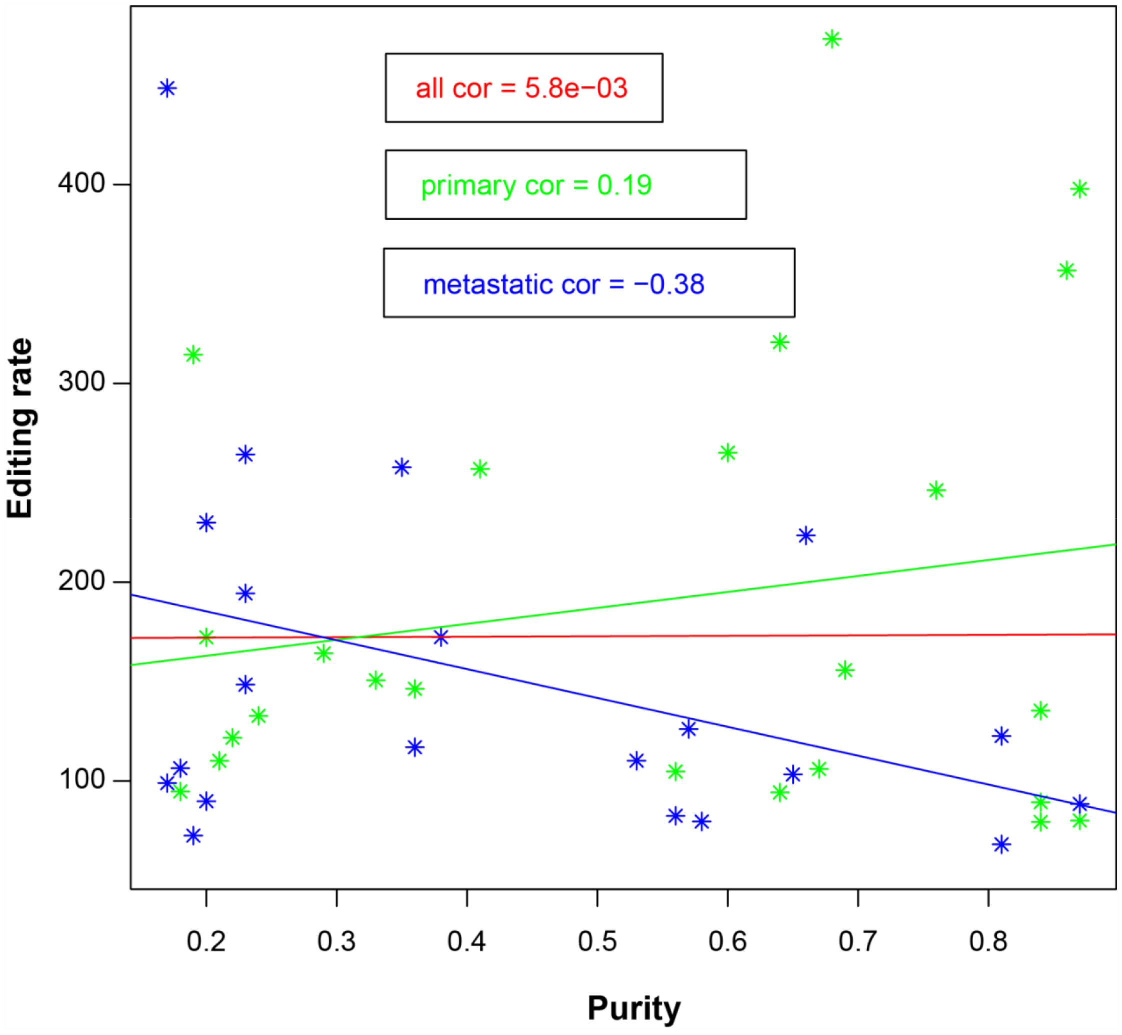

Supplementary Figure 3: The correlation between tumor purity and editing rate. Green and blue points depict the primary and metastatic samples, respectively. “all cor” means the correlation between the editing rate and purity in all samples, while “primary cor” and “metastatic cor” mean the correlation in primary and metastatic samples, respectively.

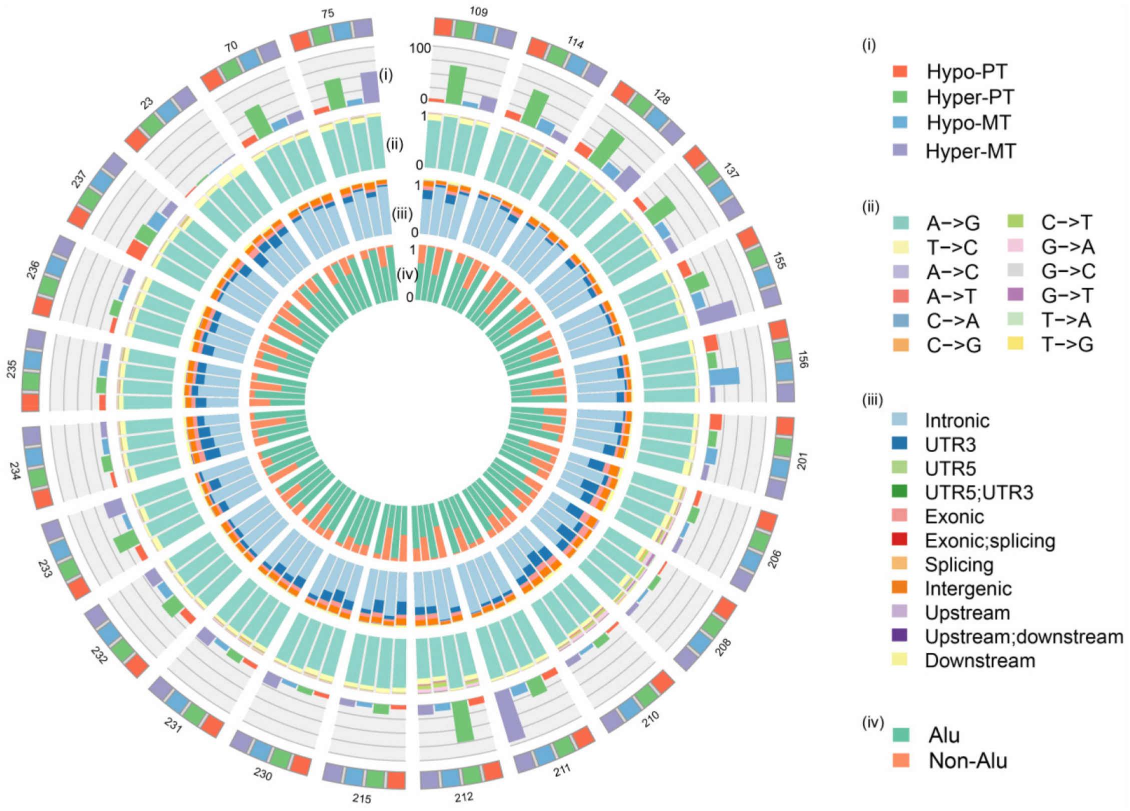

Supplementary Figure 4: Summary of hyper-editing and hypo-editing sites. Circos plot depicting the landscape of hyper/hypo-editing sites in lung adenocarcinoma. The outermost circle displays four classes of editing sites: hyper-editing sites in primary tumors (hyper-PT), hypo-editing sites in primary tumors (hypo-PT), hyper-editing sites in metastatic tumors (hyper-MT) and hypo-editing sites in metastatic tumors (hypo-MT). The numbers of the outermost circle stands for the patient ID. The (i) and (ii) circle display hyper/hypo-editing rate and the proportion of editing type for each sample, respectively. The (iii) and (iv) circle denote the distribution of hyper/hypo-editing sites in different genomic regions.

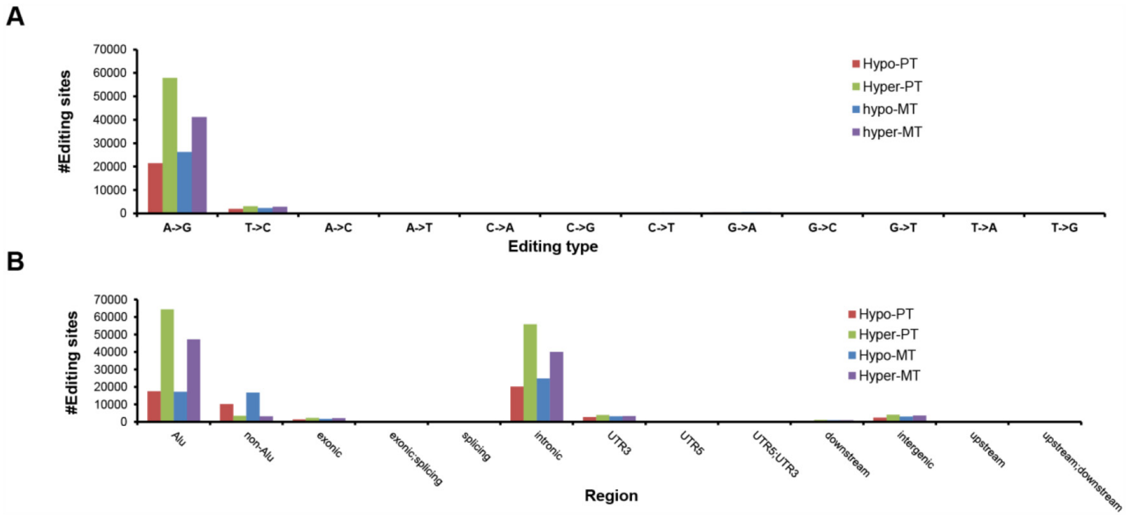

Supplementary Figure 5: Summary of editing type and the distribution of hyper/hypo-editing sites in different genomic regions. A. Histograms showing the numbers of editing types of hyper/hypo-editing sites. **B.** Histograms depicting the numbers of hyper/hypo-editing sites local in different regions across genome.

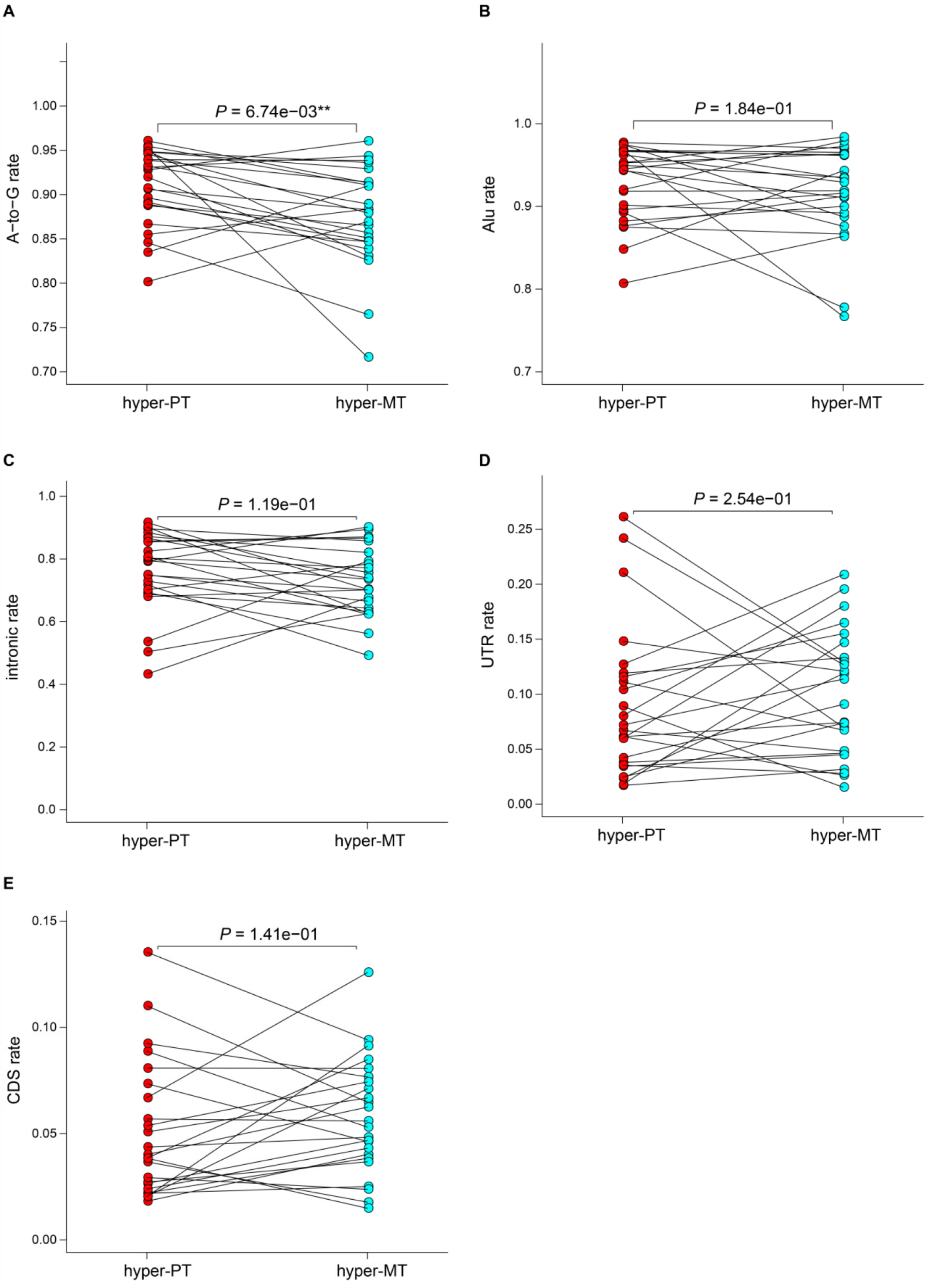

Supplementary Figure 6: The proportion of editing type or sites across different genomic regions between hyper-PT and hypo-MT. A-E. Comparison of the proportion of A -> G editing type (A), the proportion of editing events in Alu region (B) or in intronic (C) or in UTR (D) or in CDS region (E) between hyper-PT and hyper-MT.

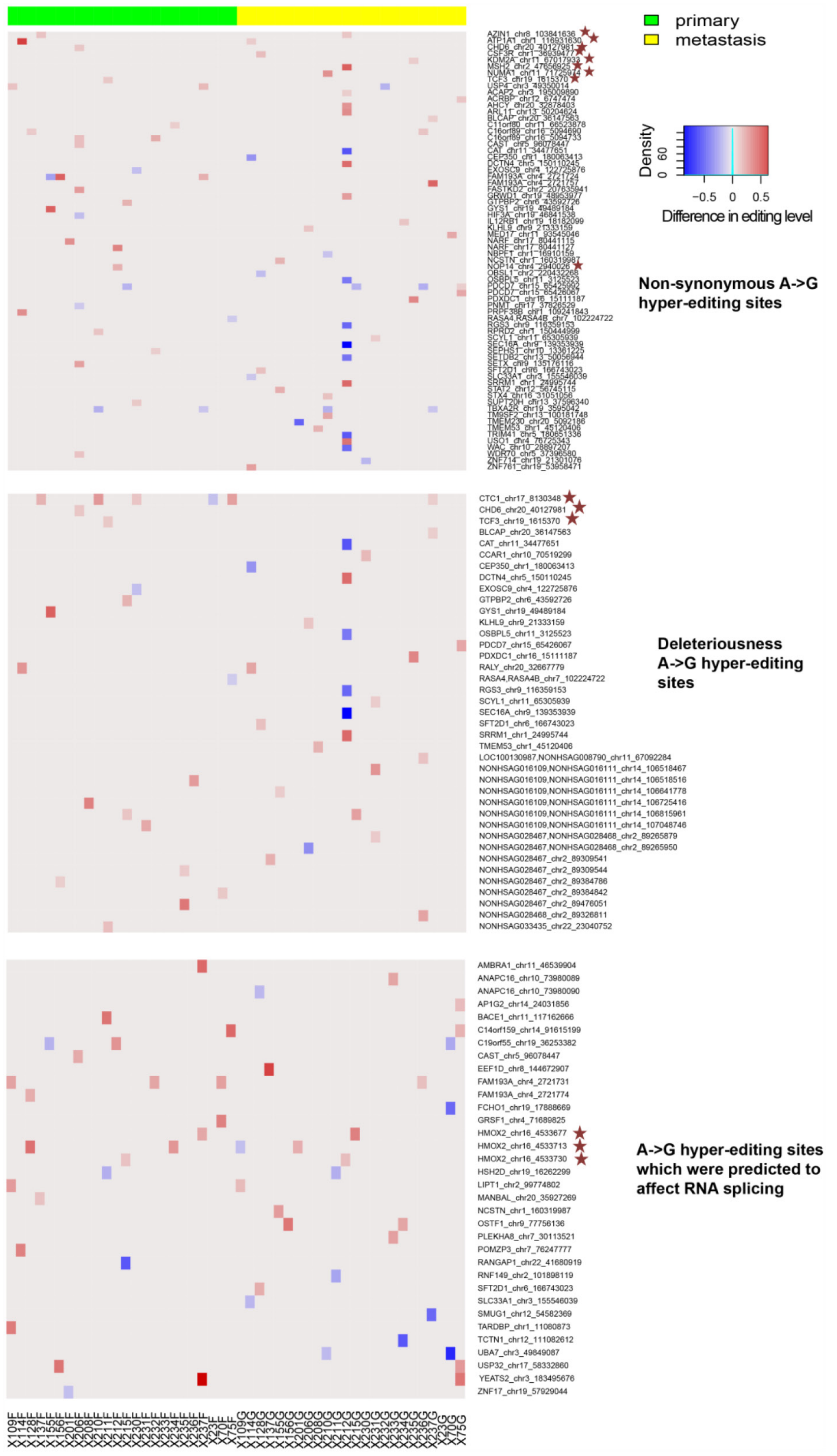

Supplementary Figure 7: The difference in editing level of selected A -> G hyper-editing sites. Starting from the top, the panels show the A -> G hyper-editing sites predicted to be non-synonymous, deleteriousness or affect RNA splicing. Genes, marked by stars, were reminded in this study, which are cancer-related genes or are reported in previous cancer studies.

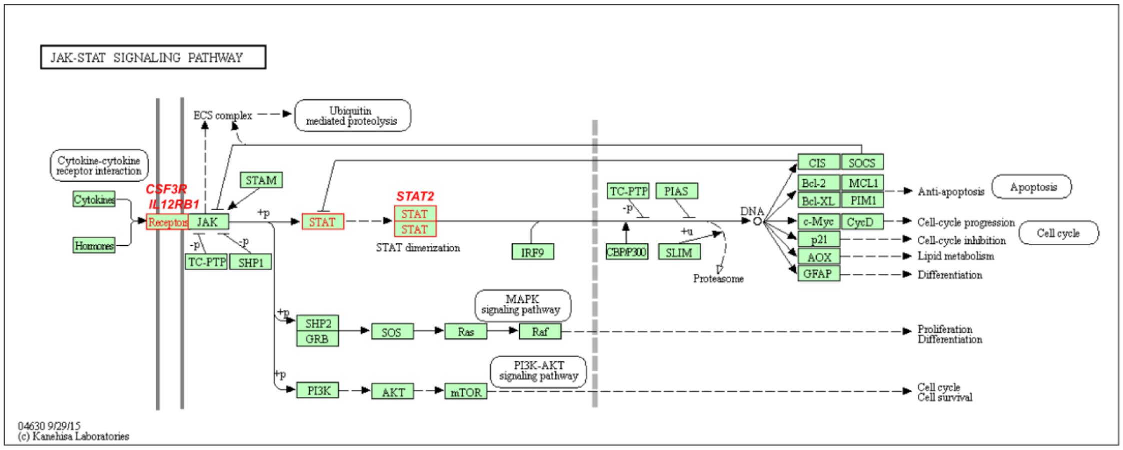

Supplementary Figure 8: Jak-STAT signaling pathway enriched in metastatic samples. 3 genes (*CSF3R*, *IL12RB1* and *STAT2*) only hyper-edited in metastatic but not in primary samples were marked in red.

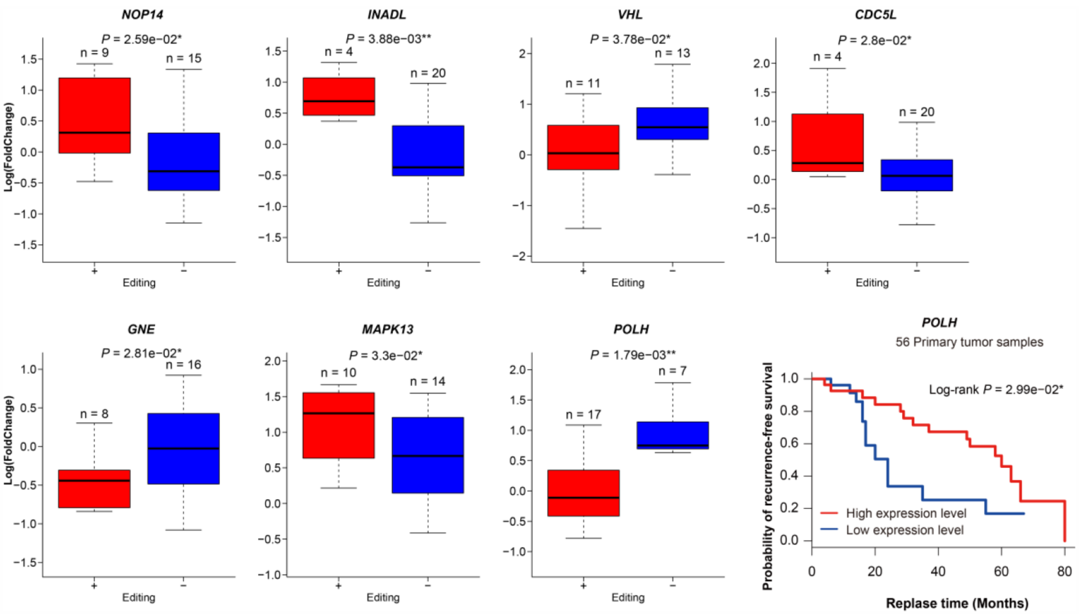

Supplementary Figure 9: Hyper-editing and gene different expression in primary samples. Seven genes with A -> G hyper-PT in UTR regions were showed. Gene different expression is presented as log (fold change). "+" or "-" indicates harboring hyper-editing sites or not in UTR region for a sample. Samples were classed to two groups: edit+ or edit-. P values are calculated by t-test. Kaplan-Meier survival curves showing the relationship of recurrence-free survival probability and *PLOH* expression.

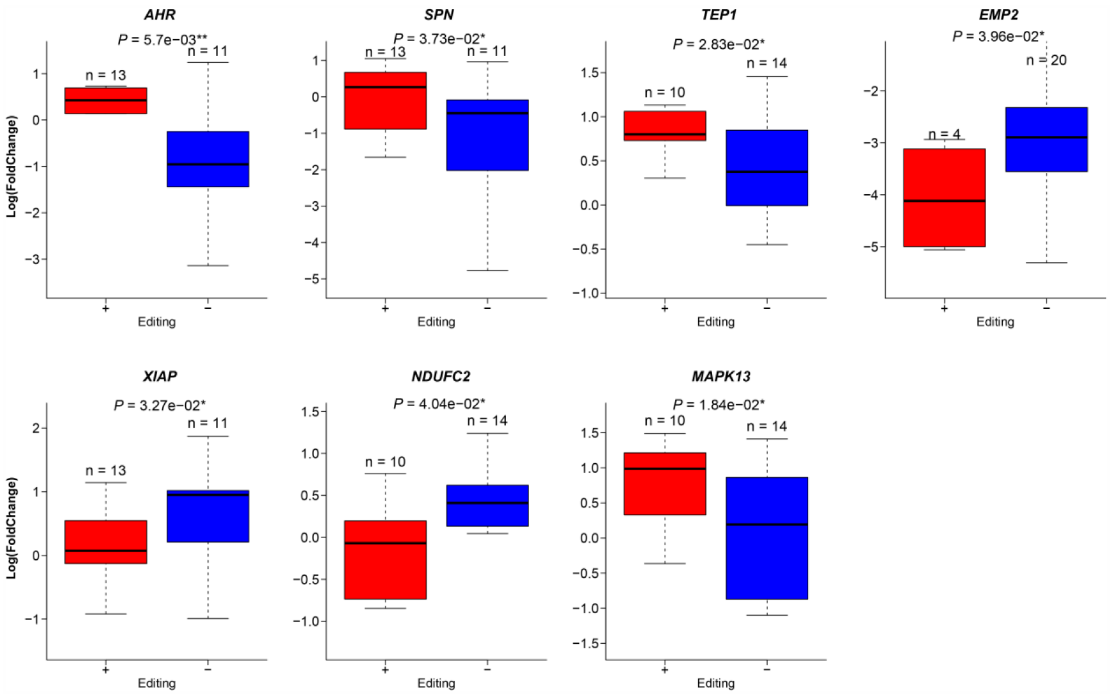

Supplementary Figure 10: Hyper-editing and gene different expression in metastatic samples. Seven genes with A -> G hyper-MT in UTR regions were showed.

Supplementary Table 1: Summary statistics of RNA edting events in each sample

See Supplementary File 1

Supplementary Table 2: The proportion of 12 RNA edting types in each sample

See Supplementary File 1

Supplementary Table 3: The distribution of RNA edting events in different regions across genome

See Supplementary File 1

Supplementary Table 4: All hyper-editing sites in primary tumors (hyper-PT)

See Supplementary File 1

Supplementary Table 5: All hypo-editing sites in primaty tumors (hypo-PT)

See Supplementary File 1

Supplementary Table 6: All hyper-editing sites in metastatic tumors (hyper-MT)

See Supplementary File 1

Supplementary Table 7: All hypo-editing sites in metastatic tumors (hypo-MT)

See Supplementary File 1

Supplementary Table 8: The genes with non-synonymous A -> G hyper-editing sites

See Supplementary File 1

Supplementary Table 9: Pathway enrichment of 30 genes with non-synonymous A -> G hyper-MT

See Supplementary File 1

Supplementary Table 10: The A->G hyper-editing sites predicted to affect RNA splicing in the human genome by SPIDEX

See Supplementary File 1

Supplementary Table 11: The deleterious A->G hyper-editing sites

See Supplementary File 1

Supplementary Table 12: The genes with significant difference in expression between two group samples: edit+ and edit-

See Supplementary File 1

Supplementary Table 13: The cancer-related gene in the literature with significant different in expression between two group samples (edit+ or edit-)

See Supplementary File 1
